# Degenerative Cervical Myelopathy in Higher-Aged Patients: How Do They Benefit from Surgery?

**DOI:** 10.3390/jcm9010062

**Published:** 2019-12-26

**Authors:** Oliver Gembruch, Ramazan Jabbarli, Ali Rashidi, Mehdi Chihi, Nicolai El Hindy, Axel Wetter, Bernd-Otto Hütter, Ulrich Sure, Philipp Dammann, Neriman Özkan

**Affiliations:** 1 Department of Neurosurgery, University Hospital Essen, University of Duisburg-Essen, 45122 Essen, Germany; 2 Spine-Center Werne, Katholisches Klinikum Lünen/Werne GmbH, St. Christophorus-Krankenhaus, Am See 1, 59368 Werne, Germany; 3 Institute of Diagnostic and Interventional Radiology and Neuroradiology, University Hospital Essen, University of Duisburg-Essen, 45122 Essen, Germany

**Keywords:** degenerative cervical myelopathy, cervical canal stenosis, cervical spine surgery, higher-aged patients, neurological outcome, mJOA Score, MCID

## Abstract

Background: Degenerative cervical myelopathy (DCM) is the most common reason for spinal cord disease in elderly patients. This study analyzes the preoperative status and postoperative outcome of higher-aged patients in comparison to young and elderly patients in order to determine the benefit to those patients from DCM surgery. Methods: A retrospective analysis of the clinical data, radiological findings, and operative reports of 411 patients treated surgically between 2007 and 2016 suffering from DCM was performed. The preoperative and postoperative neurological functions were evaluated using the modified Japanese Orthopedic Association Score (mJOA Score), the postoperative mJOA Score improvement, the neurological recovery rate (NRR) of the mJOA Score, and the minimum clinically important difference (MCID). The Charlson Comorbidity Index (CCI) was used to evaluate the impact of comorbidities on the preoperative and postoperative mJOA Score. The comparisons were performed between the following age groups: G1: ≤50 years, G2: 51–70 years, and G3: >70 years. Results: The preoperative and postoperative mJOA Score was significantly lower in G3 than in G2 and G1 (*p* < 0.0001). However, the mean mJOA Score’s improvement did not differ significantly (*p* = 0.81) between those groups six months after surgery (G1: 1.99 ± 1.04, G2: 2.01 ± 1.04, G: 2.00 ± 0.91). Furthermore, the MCID showed a significant improvement in every age-group. The CCI was evaluated for each age-group, showing a statistically significant group effect (*p* < 0.0001). Analysis of variance revealed a significant group effect on the delay (weeks) between symptom onset and surgery (*p* = 0.003). The duration of the stay at the hospital did differ significantly between the age groups (*p* < 0.0001). Conclusion: Preoperative and postoperative mJOA Scores, but not the extent of postoperative improvement, are affected by the patients’ age. Therefore, patients should be considered for DCM surgery regardless of their age.

## 1. Introduction

Degenerative cervical myelopathy (DCM) is a slowly ongoing degenerative disease of the cervical spine caused by progressive narrowing of the cervical canal and compression of the spinal cord. The DCM is age-dependent and is the most common degenerative disease of the cervical spine in elderly patients with a progressive degeneration of the intervertebral discs, joints, and ligaments. In the 4th decade of life, 30% of the population shows cervical spine degeneration and in the 6th decade about 90% suffer from cervical spine degeneration [1]. The United Nations analyzed demographic changes in the global population and showed that in 2015, 12.6% of the population worldwide was aged ≥60 years. By 2030, those people will represent 16.5% of the worldwide population and in 2050, 21.5% will be aged ≥60 years [2]. The German Census Bureau data indicates that persons above the age of 60 years are the fastest growing of all of the age groups. By 2030, 34.6% of the German population will be more than 60 years old and 37.6% of the German population will be more than 60 years old by 2050 [3]. Therefore, the frequency of patients presenting with DCM will increase. The question of surgical treatment of higher-aged patients suffering from DCM is becoming more and more important.

Previous systematic reviews have shown that non-surgical treatment in moderate to severe DCM is not recommended because of the inferior outcomes compared to surgery [4,5,6]. Fehlings et al. presented clinical practice guidelines for the management of DCM [6,7]. The cost-effectiveness of surgical treatment has also been proven in industrialized countries [8].

Surgery remains the gold standard for the treatment of DCM, but there is still no consensus among experts about therapy for higher-aged patients with DCM. There is a reluctance from surgeons to perform surgery in old patients because age is an independent factor increasing morbidity and is associated with additional comorbid medical conditions [9].

However, surgical outcomes in elderly patients with DCM are controversial. A lower surgical outcome (based on the Japanese Orthopedic Association Score (JOA Score), the neurological recovery rate (NRR), and the JOA Score improvement) in old patients has been reported in several studies [10,11,12], while other authors described no significant differences in neurological improvement between old and young patients [13,14,15].

Nevertheless, the analysis of the surgical benefit of higher-aged patients with DCM remains underrepresented in literature with respect to demographic changes.

The aim of this study was to analyze the preoperative function and the postoperative functional outcome in patients undergoing DCM surgery using the modified JOA Score (mJOA Score), the mean mJOA Score improvement, NRR, and the minimum clinically important difference (MCID) based on different age groups, with a particular focus on elderly patients.

## 2. Materials and Methods

### 2.1. Study Population

A retrospective analysis of the clinical and radiological data and operative reports of patients suffering from DCM was performed.

Data from 968 patients suffering from cervical degenerative disorders who were treated surgically in our hospital between 2007 and 2016 were analyzed applying the following exclusion criteria: 1. cervical degenerative disorders others than DCM; 2. congenital abnormalities of the cervical spine; 3. metastatic or rheumatoid diseases; 4. fractures of unknown age; 5. instability of the cervical spine; or 6. traumatic spinal cord injury.

Posterior fixation was only used in four DCM patients. Those patients were excluded from the study cohort due to the limited amount of cases. Subgroup analysis was not possible, and exclusion of those patients did not influence statistical analysis.

Therefore, 411 patients (263 males, 148 females; mean age: 62.6 ± 12.1 years; range: 31–96 years) with a spinal stenosis (249 patients, 60.6%) or a herniated disk (162 patients, 39.4%) were studied.

Patients were operated on with a ventral or posterior approach using anterior cervical discectomy and fusion (ACDF), laminoplasty, or decompressive laminectomy. Posterior fusion was not performed in DCM surgery.

**Figure 1:**
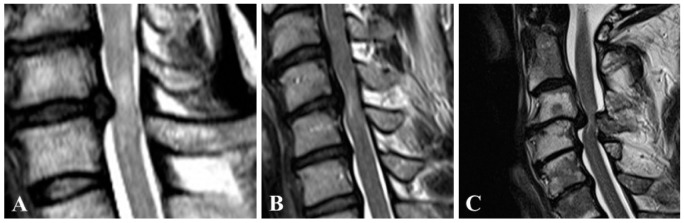
Cervical Myelopathy in a patient of G1 (**A**), G2 (**B**) and G3 (**C**).

The patients were divided into three groups depending on their age: G1: ≤50 years of age, 74 patients; G2: 51–70 years of age, 204 patients; and G3: >70 years of age, 133 patients (Figure 1). The following data were collected for all patients: age, sex, comorbidities (using the Charlson Comorbidity Index (CCI) [16], symptom presentation until surgery, preoperative status (mJOA Score) (Table 1), and the neurological outcome (mJOA Score, mean mJOA Score improvement, NRR and the MCID) (Table 2).

### 2.2. Assessment of Clinical Outcome

The severity of the DCM was evaluated before and after surgery according to mJOA Score proposed by the Japanese Orthopedic Association for cervical myelopathy [17]. The postoperative mJOA Score was assessed during the stay at the hospital, as well as three and six months after surgery. The NRR was calculated using the formula suggested by Hirabayashi and Satomi [NRR (%) = (postoperative mJOA Score−preoperative mJOA Score)/(full score (18)−preoperative mJOA Score) × 100] [18].

The mJOA Score improvement was also evaluated (postoperative mJOA Score–preoperative mJOA Score) to analyze the postoperative outcome. Comorbidities were analyzed using the CCI. A total of 27 patients (6.6%) did not attend the three-month follow-up examination and 95 patients (23.1%) were lost to follow-up six months after surgery.

Additionally, the MCID was evaluated after the operation. It is defined as the minimum change in a measurement that a patient would identify as being beneficial [19]. For DCM, the MCID is defined as follows: 1 point for patients with mild DCM (mJOA Score ≥ 15), 2 points for patients with moderate DCM (mJOA Score of 12–14), and 3 points for patients with severe DCM (mJOA Score < 12). A “poor” outcome was therefore defined as a postoperative change of mJOA Score less than the MICD.

### 2.3. Statistical Analysis

Data were analyzed using SPSS 23.0 (Statistical Package for the Social Sciences, SPSS Inc., Chicago, IL, USA). Metric data were described by mean and standard deviation and nominal data by frequency and valid percent. Data were checked for possible deviations from the assumption of normal distribution using the Shapiro-Wilk test. The mJOA Scores were assessed preoperatively, postoperatively, three months after surgery, and six months after surgery and were compared by Friedman-Tests for non-normally distributed data. The Man-Whitney-U-Test and the Kruskal-Wallis-Test were used to evaluate significant differences between the groups. The Wilcoxon signed-rank test was used to test between pairs of repeated measurement. Analysis of variance (ANOVA) was also used to detect statistical differences between the age groups. Pearson Chi^2^ statistics were applied to compute two-sided asymptotic statistical significance. In addition, a contingency coefficient served as a measure for the symmetry of the association. McNemar-Test was used within the age groups to determine significant changes in MCID improvement within a period of six months after surgery.

### 2.4. Ethics

The study has been carried out in accordance with The Code of Ethics of the World Medical Association (The Declaration of Helsinki) and was approved by the Institutional Review Board (Medical Faculty, University of Duisburg-Essen, Registration number: 16-6270-BO).

## 3. Results

### 3.1. Symptom Presentation

First symptoms in group G1 were cervicobrachial neuralgia (48.6%), followed by sensory deficits (24.3%). These results were similar to those of group G2 (43.1% and 18.6%, respectively). However, the number of patients with ataxia was higher with increasing age, particularly in G3. Here, only 30.1% of the patients suffered from cervicobrachial neuralgia as the first symptom, but 40.6% complained about ataxia (Table 3).

### 3.2. Surgical Treatment

Surgical treatment included ACDF (243 patients, 59.22%; G1: 64; G2: 130; G3: 49), laminoplasty (117 patients, 28.40%: G1: 9; G2: 61; G3: 47), and decompressive laminectomy without posterior fusion (51 patients, 17.92%; G1: 1; G2: 13; G3: 37) (Table 4).

ACDF was chosen for patients with a ventral one or two-level narrowness caused by a spinal canal stenosis or a herniated disk. In patients with multilevel spinal canal stenosis, posterior decompression was favored, while laminoplasty was performed in patients with a predominantly dorsal multilevel narrowness.

However, according to the surgical approach, the number of operated levels and the surgical treatment showed no significant MCIDs six months after the operation (Table 5).

### 3.3. Preoperative and Postoperative mJOA Score

The mean preoperative mJOA Score in G1 was 14.99 ± 2.17, the mean postoperative mJOA Score was 15.78 ± 2.22, while the mean mJOA Score three months after surgery was 16.58 ± 1.90 and the score six months after surgery was 17.25 ± 1.36.

The mean preoperative mJOA score in G2 was 14.57 ± 2.27, the mean postoperative mJOA score was 15.32 ± 2.47, while the mean mJOA score three months after surgery was 16.30 ± 1.92 and the score six months after surgery was 16.99 ± 1.39.

The mean preoperative mJOA Score in G3 was 13.57 ± 2.51, the mean postoperative mJOA Score was 14.23 ± 2.56, while mean the mJOA Score three months after surgery was 15.45 ± 2.02 and the mJOA Score six months after surgery was 16.32 ± 1.70.

**Figure 2:**
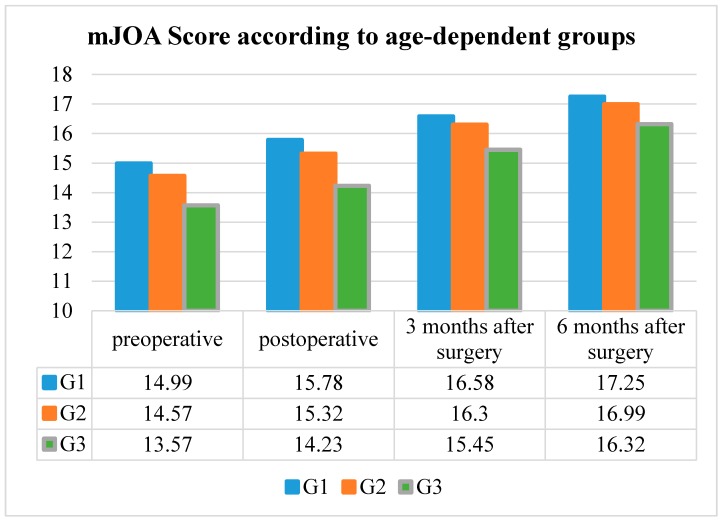
Modified Japanese Orthopedic Association Score (mJOA Score) according to the age-dependent groups (G1–G3).

The postoperative mJOA Score improved significantly (*p* < 0.001, respectively) in every age group, but higher-aged patients showed a lower postoperative mJOA Score compared to younger and elderly patients (Table 2 and Figure 2).

Additionally, postoperative mJOA Scores increased significantly independently of the surgical procedure (Table 6).

### 3.4. Neurological Recovery Rate

The median NRR in patients according to G1 was 37.6% postoperative, 65.2% three months after surgery, and 83.4% six months after surgery in patients G1.

The median NRR in patients according to G2 was 32.9% postoperative, 60.3% three months after surgery, and 75.8% six months after surgery in patients G2.

The median NRR in patients according to G3 was 19.5% postoperative, 46.2% three months after surgery, and 61.9% six months after surgery.

**Figure 3:**
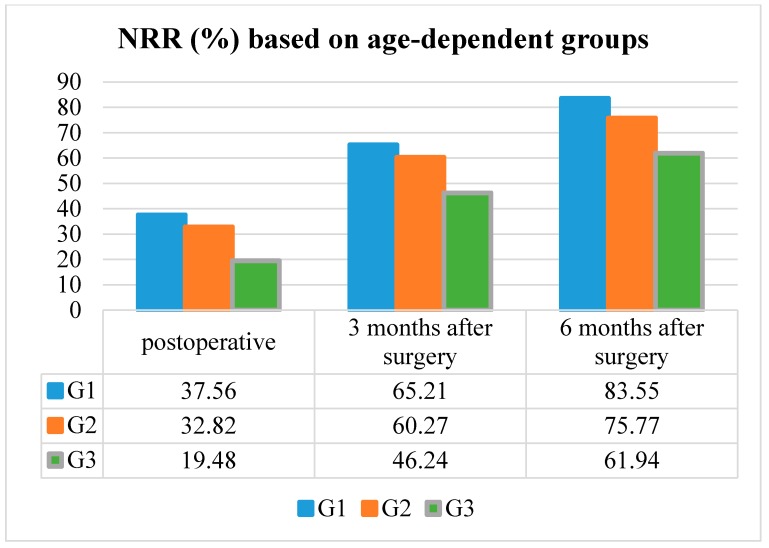
The neurological recovery rate (NRR) based on the age-dependent groups.

NRR improved significantly in every age group at the postoperative, three and six months post-surgery follow-up examinations (*p* < 0.001, respectively). Additionally, patients belonging to G1 or G2 presented a significantly better recovery rate than patients representing G3, due to the higher preoperative mJOA Scores (*p* < 0.001) (Table 2 and Figure 3).

Additionally, postoperative NRR increased significantly independently of the surgical procedure used (Table 6).

### 3.5. Mean mJOA Score Improvement

The mean mJOA Score improvement in G1 was 0.76 ± 0.79 postoperative, 1.58 ± 0.90 three months after surgery, and 1.95 ± 1.04 six months after surgery.

The mean mJOA Score improvement in G2 was 0.74 ± 0.97 postoperative, 1.60 ± 0.89 three months after surgery, and 2.01 ± 1.04 six months after surgery.

The mean mJOA Score improvement in G3 was 0.66 ± 1.02, 1.58 ± 1.13 three months after surgery, and 2.00 ± 0.91 six months after surgery.

**Figure 4:**
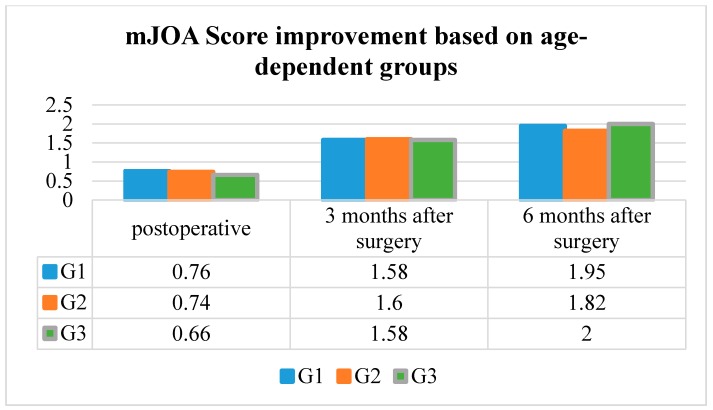
mJOA Score improvement based on the age-dependent groups.

The mean mJOA Score improvement was significantly better in every age group at the postoperative, three and six months post-surgery follow-up examinations (*p* < 0.001, respectively). However, the mean mJOA Score improvement did not differ significantly between the age groups (*p* = 0.81) (Table 2 and Figure 4).

Additionally, postoperative mJOA Score improvement increased significantly independently of the surgical procedure used (Table 6).

### 3.6. Minimum Clinically Important Difference

In our study, we were able to show favorable MCID immediately postoperatively in 52.7% of the patients of G1, in 49.0% of the patients of G2, and in 34.6% of the patients of G3. Furthermore, 98.4% (G1), 93.3% (G2), and 85.7% (G3) of the patients achieved favorable MCID six months after surgery. MCID was significantly within the different age groups. Unfortunately, the MCID achievements were significantly reduced in elderly patients compared to younger patients (Table 2).

Furthermore, the MCID six months postoperative revealed no significant differences regarding the different surgical approaches (ventral vs. dorsal), the number of operated levels (monosegmental, bisegemental, and multisegmental), and the different surgical treatment (ACDF, laminoplasty and laminectomy) (Table 5).

### 3.7. Comorbidities and the Charlson Comorbidity Index

Comorbidities of the patients were collected and grouped as 1. cardiovascular (e.g., arterial hypertension, coronary heart disease myocardial infarction), 2. pulmonal (e.g., chronic obstructive lung disease, pneumonia, asthma bronchiale), 3. neurological (e.g., transitory ischemic attack, stroke, polyneuropathia), 4. oncological (e.g., lung cancer, breast cancer or prostate cancer), 5. endocrine disease (e.g., thyreoditis, diabetes mellitus), and 6. surgical (abdominal, cardial, pulmonal or orthopedic).

The CCI was evaluated for each age group, showing a statistically significant group effect (*p* < 0.001) according to ANOVA. Additionally, CCI showed a significant association with the preoperative and postoperative mJOA Score (*p* < 0.001) (Table 1).

### 3.8. Duration of Myelopathic Symptoms Prior to Surgery

In our study, the duration lasting from the first symptom presentation until DCM surgery was much longer in G3 (45.9 ± 66.0 weeks; range: 1–350) than in G1 (22.2 ± 22.08 weeks; range: 1–122) and G2 (33.3 ± 43.6 weeks; range: 1–300). ANOVA revealed a significant group effect on the delay (weeks) between symptom onset and surgery (*p* = 0.003) (Table 1).

### 3.9. Duration of Hospitalization

Stay at the hospital in G1 was around 8.6 ± 3.8 days, 9.6 ± 4.0 days in G2, and 10.5 ± 4.7 days in G3. The duration of the stay at the hospital did differ significantly (*p* < 0.001) between the age-groups according to the ANOVA results (Table 1).

### 3.10. Surgical and Non-Surgical Complications

Complications were analyzed according to the age groups. Surgical and non-surgical complications were evaluated. Surgical complications were defined as 1. postoperative bleeding, 2. poor wound healing, 3. cerebrospinal fluid leakage, and 4. acute myelon compression. Non-surgical complications were defined as 1. pneumonia, 2. heart attack, and 3. stroke.

Non-surgical complications were highest in G3 with 6.0%, whereas those complications were similar within G1 and G2 (1.4% vs. 1.5%).

Surgical complications were reported to increase with age, affecting 1.4% (G1), 2.5% (G2), and 6.8% (G3) of the patients (Table 4).

## 4. Discussion

The demographic changes of the population especially in western countries [3] have led to an increase of age-dependent diseases. Therefore, understanding the treatment of elderly patients has become more relevant. At present, there are no guidelines for management of degenerative spine diseases in elderly individuals. Moreover, only a few articles have addressed this topic so far, returning partially discrepant results in functional outcomes [10,11,12,13,14,15].

Tetreault et al. showed in their systematic review that patients with a more severe DCM expressed by a lower mJOA Score and patients with a longer duration of the symptoms are more likely to have a worse surgical result. They concluded that both severe and chronic compression of the spinal cord may lead to irreversible damage due to demyelination and necrosis of the grey matter. They were also able to show that age is a potential predictor when analyzing the postoperative outcome [20]. Holly et al. also analyzed age, the duration of symptoms, and preoperative neurological function as predictors of the neurological outcome and they could show similar results in their review [21]. In our study, the time between the first symptom presentation and DCM surgery was much longer in G3 than in G1 or G2. This effect might be caused by the fact that patients in G3 suffer from more comorbidities than younger patients and that an age-dependent decrease in the daily condition might be seen as normal or might not be recognized early in older patients and therefore, the time until diagnosis is prolonged. The reduced physical condition is also expressed by the prolonged hospitalization in G3 compared to G1 and G2 and the significantly lower CCI. The CCI also showed a strong correlation with the preoperative and postoperative mJOA Scores. Additionally, the first symptom of G1 patients was cervicobrachial neuralgia, whereas the first symptom of G3 patients was ataxia followed by cervicobrachial neuralgia. This could highlight the difficulties of distinguishing DCM from other age-related diseases.

The evaluation of the preoperative mJOA Score showed significantly lower scores in G3 compared to the preoperative mJOA scores of G1 and G2. The postoperative mJOA Score and the mJOA Score three and six months after surgery showed similar results.

In our study, the significantly lower preoperative and postoperative mJOA Scores of G3 patients as compared to G2 and G1 patients was strongly associated with the lower CCI of those patients. Mean preoperative and postoperative mJOA Scores may be lower in G2 and especially in G3 due to physical weakness caused by age and known comorbidities such as cerebral vascular disorders, hip and knee osteoarthritis, entrapment of peripheral neuropathy (carpal or cubital tunnel syndrome), diabetic neuropathy, benign prostatic hypertrophy, or urinary stress incontinence [22]. Machino et al. [23] also concluded that the preoperative JOA Score might be influenced by those comorbidities.

Nagashima et al. evaluated the neurological outcomes of 37 patients over 80 years of age and compared them with that of a younger population. The NRR was lower in the elderly population, but JOA Scoring improved in a way that life style was positively influenced [10].

In the present study, there was a significant improvement according to the NNR after surgery in all age groups. Interestingly, NRR was significantly lower in G3 compared to G1 and G2 despite similar mean mJOA Score improvements.

Nevertheless, there are limitations concerning the validity of the NRR despite its popularity. The results of the NRR are strongly influenced by the preoperative mJOA Scores. For example, if patients have a low preoperative mJOA Score, then NRR is lower than in patients with a higher preoperative mJOA Score even though the mean mJOA Score improvements are the same [23].

Due to the limitations of the NRR evaluating the neurological outcome in patients with a lower preoperative mJOA Score, the mean mJOA Score improvement or the MCID might be more valuable for comparing the neurological improvements between those patients.

In our study, the mean mJOA Score improvements were similar in every age-dependent group (Table 3), although the preoperative mJOA Score was significantly lower in G3 than in G1 or G2.

The results of the mean mJOA Score improvement are in line with the findings of Machino et al. [23]. They analyzed 520 patients with CSM treated by laminoplasty and divided their patients into nonelderly (<65 years), young-old (65–75 years) and old-old (>75 years). Elderly group patients showed significantly lower recovery rates of JOA Scores compared with the nonelderly group, but mean JOA Score improvements showed no difference among these groups. Preoperative JOA Scores were also significantly lowered similarly to our patients.

Madhavan et al. also performed a meta-analysis of old DCM patients evaluating the postoperative outcome and the operative risks. They found, like in our evaluation, a significantly lower preoperative JOA Score and a lower postoperative JOA Score associated with a lower NRR. But postoperative and long-term improvements in old patients have been remarkable in terms of improvements in mobility and independence, leading to reduced nursing care being required. The incidence of postoperative complications did not show a significant difference [24].

Additionally, MCID was favorable in the majority of elderly patients (85.7%) six months after surgery. This means that those patients showed acceptable clinical improvement after surgery despite their age.

In summary, mean mJOA Score improvements did not differ significantly among the age-dependent groups, but clinical improvement after surgery according to the mJOA Score was much better in old patients compared to younger patients. This improvement led to an improvement in mobility and independence, hence requiring reduced nursing care. This was shown by Yoshida et al., who were able to show in a study with 76 patients older than 75 years of age that the nursing care requirements based on JOA Score and functional independence measure scoring was reduced [11]. Furthermore, a different surgical approach, number of operated levels, and surgical treatment revealed no significant MCID differences six months post-surgery.

### Limitations

The present study has several limitations. First, this is a retrospective, non-randomized study with the associated inherent bias. The analyzed data were collected from documented electronic records, operative reports, radiological data, and reports of the patients. Secondly, the mJOA Scoring system might be influenced in the elderly group by several comorbidities such as hip and knee osteoarthritis, cerebrovascular diseases, diabetic neuropathy, or prostate hypertrophy. Additionally, the NRR system also has some limitations. Lower preoperative scores indicate lower NRR, although the mean mJOA Score improvement was the same. Furthermore, the follow-up period of six months is relatively short, caused by the retrospective nature of the study and the resulting losses in follow-up. The evaluation of the postoperative outcome might be too early directly after surgery, but we were still able to show an improvement of the neurological status over that short period.

However, future prospective studies with a longer follow-up are needed to evaluate the neurological long-term outcome in elderly patients. Nevertheless, we could show in a large population of elderly patients that surgery is still useful due to clinical improvements of the symptoms and the resulting lower need for daily care.

## 5. Conclusions

The preoperative and postoperative mJOA Scores are significant lower in older patients compared to younger individuals, but the mean mJOA Score improvement is similar. The lower mJOA Score in those patients correlates with a lower CCI. With respect to the postoperative mJOA Score improvement and MCID, older patients still benefit from surgery. Therefore, surgical treatment of DCM is a valuable option for those patients.

## Figures and Tables

**Table 1:** Clinical parameters of the treated patients; m: male; f: female.

	G1	G2	G3	*p*-Value
Median Age	44.45 ± 4.57	60.34 ± 5.61	75.66 ± 5.06	-
Male	42	144	77	-
Female	32	60	56	-
Charlson Comorbidity Index (%)	95.7 ± 11.6	86.5 ± 13.5	50.8 ± 26.2	*p* < 0.001
Symptom duration (weeks)	22.19 ± 22.08	33.3 ± 43.62	45.88 ± 66.02	*p* = 0.003
Stay at the hospital (days)	8.6 ± 3.8	9.6 ± 4.0	10.5 ± 4.7	*p* < 0.001

**Table 2:** Modified Japanese Orthopedic Association Score (mJOA Score), neurological recovery rate (NRR) and the mean mJOA Score improvement according to the age groups (G1–G3) postoperative, three and six months after surgery.

Neurological Outcome	G1	G2	G3	*p*-Value
mJOA Score preoperative	14.99 ± 2.17	14.57 ± 2.27	13.57 ± 2.51	*p* < 0.001
mJOA Score postoperative	15.78 ± 2.22	15.32 ± 2.47	14.23 ± 2.56	*p* < 0.001
mJOA Score 3 months postoperative	16.58 ± 1.90	16.30 ± 1.92	15.45 ± 2.02	*p* < 0.001
mJOA Score 6 months postoperative	17.25 ± 1.36	16.99 ± 1.39	16.32 ± 1.69	*p* < 0.001
NRR (%) postoperative	37.6	32.9	19.5	*p* < 0.001
NRR (%) 3 months postoperative	65.2	60.3	46.2	*p* < 0.001
NRR (%) 6 months postoperative	83.4	75.8	61.9	*p* < 0.001
Mean mJOA Score improvement postoperative	0.76 ± 0.79	0.74 ± 0.97	0.66 ± 1.02	*p* = 0.186
Mean mJOA Score improvement 3 months postoperative	1.58 ± 0.90	1.60 ± 0.89	1.58 ± 1.13	*p* = 0.948
Mean mJOA Score improvement 6 months postoperative	1.95 ± 1.04	2.01 ± 1.04	2.00 ± 0.91	*p* = 0.835
MCID postoperative	39/74 (52.7%)	100/204 (49.0%)	46/133 (34.6%)	*p* = 0.011
MCID 3 months postoperative	64/72 (88.9%)	155/192 (80.7%)	86/120 (71.7%)	*p* = 0.014
MCID 6 months postoperative	60/61 (98.4%)	153/164 (93.3%)	78/91 (85.7%)	*p* = 0.012

**Table 3:** Analyzing the first presenting symptom in relation to the age groups.

First Symptom	G1	G2	G3
Cervicobrachial neuralgia	36 (48.6%)	88 (43.1%)	40 (30.1%)
Sensory deficit	18 (24.3%)	38 (18.6%)	23 (17.3%)
Paresis	5 (6.8%)	17 (8.4%)	16 (12.0%)
Ataxia	15 (20.3%)	61 (29.9%)	54 (40.6%)

**Table 4:** Surgical treatment and complications according to the age groups; ACDF: Anterior cervical discectomy and fusion.

		G1	G2	G3
**Surgical Treatment**	ACDF	64	130	49
	Laminoplasty	9	61	47
	Laminectomy	1	13	37
**Complications**	Surgical	1/74 (1.4%)	5/204 (2.5%)	9/133 (6.8%)
	Non-surgical	1/74 (1.4%)	3/204 (1.5%)	8/133 (6.0%)

**Table 5:** The minimum clinically important difference (MCID) six months after surgery according to the surgical approach, the number of operated levels, and the surgical treatment; anterior cervical discectomy and fusion: ACDF.

		MCID Achievement 6 Months Postoperative		*p*-Value
		Yes	No	
**Approach**	ventral	181	14	*p* = 0.669
	dorsal	110	11	
**Number of operated levels**	monosegmental	162	11	*p* = 0.521
	bisegmental	69	7	
	multisegmental	60	7	
**Surgical treatment**	ACDF	178	14	*p* = 0.282
	Laminoplasty	84	6	
	Laminectomy	29	5	

**Table 6:** Modified Japanese Orthopedic Association Score (mJOA Score), neurological recovery rate (NRR) and the mean mJOA Score improvement according to the surgical treatment; anterior cervical discectomy and fusion: ACDF.

	Surgical Treatment	Preoperative	Postoperative	3 Months Postoperative	6 Months Postoperative	*p*-Value
**mJOA Score**	ACDF	14.8 ± 2.3	15.6 ± 2.4	16.6 ± 1.7	17.2 ± 1.3	<0.001
	Laminoplasty	13.9 ± 2.4	14.7 ± 2.4	15.7 ± 2.2	16.6 ± 1.6	<0.001
	Laminectomy	12.9 ± 2.3	13.8 ± 2.2	14.9 ± 1.8	15.7 ± 1.7	<0.001
**mJOA Score improvement**	ACDF		0.8 ± 06	1.5 ± 0.8	1.9 ± 1.0	<0.001
	Laminoplasty		0.8 ± 0.7	1.8 ± 0.9	2.3 ± 0.9	<0.001
	Laminectomy		0.8 ± 0.8	1.8 ± 1.0	2.1 ± 0.8	<0.001
**NRR**	ACDF		33.8 ± 33.6	61.9 ± 33.4	76.9 ± 30.2	<0.001
	Laminoplasty		25.0 ± 26.9	53.6 ± 31.4	71.6 ± 27.0	<0.001
	Laminectomy		21.4 ± 24.6	41.6 ± 23.5	54.1 ± 24.5	<0.001
